# Nanoparticles loaded with IL-2 and TGF-β promote transplantation tolerance to alloantigen

**DOI:** 10.3389/fimmu.2024.1429335

**Published:** 2024-07-26

**Authors:** David A. Horwitz, Ju Hua Wang, Dongin Kim, Chang Kang, Katja Brion, Sean Bickerton, Antonio La Cava

**Affiliations:** ^1^ General Nanotherapeutics, Santa Monica, CA, United States; ^2^ Department of Medicine, Keck School of Medicine, University of Southern California, Los Angeles, CA, United States; ^3^ Department of Pharmaceutical Sciences, University of Oklahoma Health Sciences Center, Oklahoma City, OK, United States; ^4^ Department of Medicine, University of California, Los Angeles, Los Angeles, CA, United States; ^5^ Department of Biomedical Engineering, Yale University, New Haven, CT, United States; ^6^ Department of Medicina Molecolare e Biotecnologie Mediche, Federico II University of Naples, Naples, Italy

**Keywords:** (NP) nanoparticle, (GVHD) graft-versus-host disease, (MLR) mixed lymphocyte reaction, (DC) dendritic cell, (Treg) T regulatory cell

## Abstract

We have previously reported that nanoparticles (NPs) loaded with IL-2 and TGF-β and targeted to T cells induced polyclonal T regulatory cells (Tregs) that protected mice from graft-versus-host disease (GvHD). Here, we evaluated whether administration of these NPs during alloantigen immunization could prevent allograft rejection by converting immunogenic responses to tolerogenic ones. Using C57BL/6 mice and BALB/c mice as either donors or recipients of allogeneic splenocytes, we found that treatment with the tolerogenic NPs in both strains of mice resulted in a marked inhibition of mixed lymphocyte reaction (MLR) to donor cell alloantigen but not to third-party control mouse cells after transfer of the allogeneic cells. The decreased alloreactivity associated with a four- to fivefold increase in the number of CD4^+^ and CD8^+^ T regulatory cells (Tregs) and the acquisition of a tolerogenic phenotype by recipient dendritic cells (DCs) in NP-treated mice. As allogeneic cells persisted in NP-treated mice, these findings suggest that tolerogenic NPs can induce alloantigen-specific Tregs and tolerogenic DCs promoting tolerogenic responses to alloantigen. By inhibiting reactivity to allotransplant, this approach could help reduce the need for immune suppression for the maintenance of allografts.

## Introduction

Although the use of immunosuppressive drugs for the prevention of allogeneic transplant rejection has resulted in increased long-term transplant survival, it carries adverse side effects in the host because of the non-specific, global immune suppression. To overcome this problem and to induce immune tolerance to transplanted organs with limited side effects from immune suppression, T regulatory cells (Tregs) have been used for the achievement of graft survival without immunosuppressive drugs (1) via adoptive immunotherapy of genetically engineered Tregs or with Tregs expanded *ex vivo (* 2–5 *).*

However, the use of autologous polyclonal Tregs or donor-reactive CD4^+^ Tregs in clinical transplantation trials has generally been only mildly encouraging, with the exception of one study that suggested the possibility to reduce or discontinue immune suppression after liver transplantation (6).

Several strategies have been developed to induce a tolerogenic environment *in vivo*. One is to induce functionally stable tolerogenic CD4^+^ Tregs based on the knowledge on IL-2, TGF-β, and continuous T-cell receptor (TCR) stimulation (7). Alternatively, Tregs can be expanded *in vivo* using low-dose IL-2, IL-2/anti-IL-2 complexes, or IL-2 muteins, as demonstrated in clinical trials (8–10). The use of antibodies blocking T-cell coreceptors and costimulatory molecules to enhance CD4^+^ Tregs has instead not reached the clinic due to the associated adverse side effects (11).

To induce tolerogenic immune responses *in vivo*, we have used biodegradable poly(lactic-co-glycolic acid) (PLGA) nanoparticles (NPs) decorated with anti-CD2 antibodies (Ab) and encapsulating IL-2 and TGF-β to target T cells for induction of CD4^+^ and CD8^+^ Tregs (12). We assessed here the potential of this approach to also induce transplantation tolerance for several reasons. First, the treatment with anti-CD2 antibodies is known to prolong allograft survival (13). Second, our NPs induced NK cells to produce TGF-β, which is tolerogenic (14). Third, our NPs use nanomolar doses of IL-2 with equivalent effects to soluble IL-2 administered systemically at a 1,000-fold higher concentration (15). Fourth, the NP therapeutic efficacy has already been proven in murine GvHD (16, 17). Fifth, the protection from disease conferred by the NPs associates with induction of both CD4^+^ and CD8^+^ Tregs (16, 17), which play a major role in transplantation tolerance (3).

To address the above possibility, we studied the NP effects in mice immunized with MHC-mismatched splenocytes. We found that treatment with the NPs converted immunogenic responses to alloantigen to tolerogenic responses with 1) a marked reduction of mixed lymphocyte response (MLR) to donor alloantigen; 2) the expansion of CD4^+^ and CD8^+^ Tregs, 3) the induction of tolerogenic dendritic cells (DCs), and 4) the persistence of allogeneic cells in NP-treated mice. Specificity of the tolerogenic response to alloantigen was indicated by the lack of an inhibition of the MLR in third-party controls.

These results imply that when the donor allograft is known, the conditioning of the recipient with donor alloantigen together with tolerogenic NPs can allow the maintenance of the graft, in our case without a concomitant use of immunosuppressive drugs.

## Methods

### Mice

8-to-10-week-old female C57BL/6 (H2^b^), BALB/c (H2^d^), and MRL (H2^k^) mice were used as donor of splenocytes for the adoptive transfer experiments. All mice were purchased from the Jackson Laboratory (Bar Harbor, ME) and were housed under pathogen-free conditions on a 12-h light/dark cycle, allowed to *ad libitum* food and water. Their use followed guidelines approved by the Institutional Animal Care and Use Committee (IACUC) under IACUC-approved protocols and in compliance with the *Guide for the Care and Use of Laboratory Animals, 8^th^ Ed.* (National Research Council (US) Committee, 2011).

### Nanoparticles

Avidin-coated PLGA NPs were prepared and characterized for physical properties assessing encapsulation metrics and release kinetics according to previously published protocols (14, 16, 17). A schematic diagram of NP assembly is shown in **Supplementary Figure 1**. The dynamic light scattering indicated that the NPs had a mean ± SD hydrodynamic diameter of 268.5 ± 12.7 nm with a low polydispersity index and a relatively tight size distribution. The encapsulation of the cytokines in the NPs was measured by ELISA after disruption of the NPs with DMSO. Standard curves were generated using known cytokine concentrations. The mean content ± SD of IL-2 and TGF-β in the NPs was 11.1 ± 2.2 ng of TGF-β and 2.4 ± 1.2 ng IL-2 per mg of NP. For cell targeting, NPs were diluted in phosphate-buffered saline (PBS) and incubated for 10 min with 5-µg biotinylated anti-CD2 antibody (clone RM2-5, Thermo Fisher Scientific, Waltham, MA)/mg NP (18). Because of our past studies, we refer hereafter to these anti-CD2 antibody-decorated NPs containing IL-2 and TGF-β as tolerogenic NPs.

### Optimization of nanoparticle dosing

Groups of mice (n = 6–8 per group) were administered a total of 7 mg of tolerogenic NPs i.p. over a time frame of 7 days. Specifically, one group received 1 mg NPs every day, one group received 2.3 mg on alternate days, and another group received 3.5 mg every 3 days. Controls only received PBS. Percentages of circulating CD4^+^ and CD8^+^ (Foxp3^+^) Tregs were assessed *ex vivo* by flow cytometry (see below) on days 0, 7, 14, and 21.

### Cell preparation

Peripheral blood mononuclear cells (PBMCs) were purified from heparinized venous blood according to published procedures (19). Spleens were aseptically removed from euthanized mice and single-cell suspensions prepared by passage through a presoaked nylon mesh. Red blood cells were removed using Tris-buffered NH_4_Cl solution. After washes and determination of cell viability by trypan blue dye exclusion, 1 × 10^7^ splenocytes diluted in PBS were injected i.v. into the recipient mice.

### Alloimmunization protocol

C57BL/6 (H2^b^) and BALB/c (H2^d^) mice were used as either donor or responder in adoptive transfer studies of mouse splenocytes. Five groups of mice (n = 8 each) were immunized with allogeneic splenocytes. Group 1 (allo only) also received a boost of allogeneic cells on day 24. Group 2 (NP-primed) received tolerogenic NPs daily from day 0 to day 6. Group 3 (NP-primed + NP boost) received NPs daily from day 0 to day 6 and then additional NPs (1 mg/dose) every 3 days thereafter, until day 27. Group 4 (NP-primed + allo boost) received NPs daily from day 0 to day 6 and a boost with allogeneic cells on day 24. Group 5 was similar to group 3 with the difference that also received a boost with allogeneic cells on day 24. MLR to third-party control mice (MRL mice, H2^k^) were performed on days 15 and 30. The frequency of Tregs was evaluated *ex vivo* by flow cytometry on days 0, 7, 14, 21, and 28 and dendritic cell phenotype on day 14.

### Mixed lymphocyte reaction

1 × 10^5^ “responder” PBMCs were prelabeled for 10 min with 0.5 μM carboxyfluorescein diacetate succinimidyl ester (CFSE) (Thermo Fisher Scientific) (0.5 µM CFSE at 37°C for 10 min) were cocultured in 96-well round-bottom plates (Corning Life Sciences, Lowell, MA) together with T-cell-depleted splenocytes (positively selected using microbeads on autoMACS^®^ [Miltenyi Biotec, Gaithersburg, MD]) serving as allogeneic “stimulators”. The stimulator-to-responder cell ratio was 1:4. After 72 h of coculture at 37°C and 5% CO_2_, flow cytometry evaluated CSFE staining. Control wells contained individual (non-mixed) cells.

### Flow cytometry

Phenotypic analyses were performed with combinations of fluorochrome-conjugated monoclonal antibodies (Ab) using standard techniques. After Fc blocking, fluorochrome-conjugated Ab to CD4 (clone GK1.5), CD8 (clone 53-6.7), CD25 (clone PC61.5), H-2^d^ (clone 34-1-2S), or isotype control was used for surface staining of T cells. Intracellular staining for Foxp3 was done using the eBioscience™ Mouse Regulatory T Cell Staining kit (Thermo Fisher Scientific) according to the manufacturer’s instructions. For the DCs, fluorochrome-conjugated Ab to the following markers were used: anti-CD11c (clone N418), anti-H2 I-A (clones M5/114.15.2 to H2^b^ and 17-5323-82 to H2^d^), anti-CD80 (clone 16-10A1), and anti-CD86 (clone GL1). All antibodies were from Thermo Fisher Scientific. Stained cells were acquired on a FACSCalibur™ flow cytometer (BD Biosciences, San Jose, CA) and analyzed using FlowJo software (Tree Star Inc., Ashland, OR).

### Statistics

Normal distribution was assessed by the Shapiro–Wilk test. Comparisons used the Student’s t test between two groups and one-way ANOVA with Bonferroni’s correction for multiple groups. Data analysis was done using GraphPad (Prism Software, Irvine, CA). Results are reported as mean + SEM. P values <0.05 were considered significant.

## Results

### Effects of tolerogenic NPs on allogeneic T-cell responses

**Figure 1:**
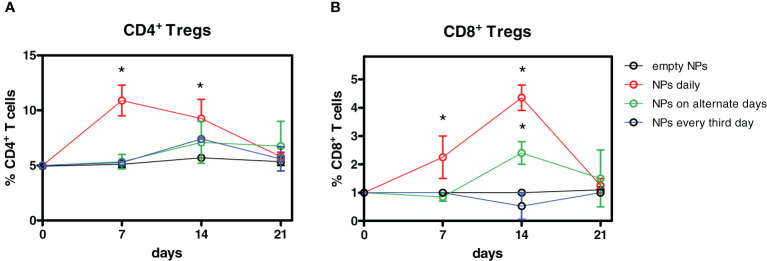
Optimization of the timing of administration of tolerogenic nanoparticles (NPs) for the induction of Tregs. Four groups of mice (n = 6 each) were injected i.p. with a total of 7 mg anti-CD2 Ab-coated NPs containing IL-2 and TGF-β over 7 days. One group received 1 mg NPs daily, one group received 2.3 mg NPs on alternate days, and another group received 3.5 mg NPs every 3 days. Controls received empty NPs. Weekly *ex vivo* blood monitoring by flow cytometry identified a peak expansion of CD4^+^ Tregs by day 7 and by day 14 for CD8^+^ Tregs. Results are from two independent experiments; *P < 0.05 by ANOVA vs. empty NPs.

To determine the optimal conditions of delivery of the tolerogenic NPs for the induction of Tregs, mice were injected i.p. with a total of 7 mg of NPs at varying intervals (ranging from every day to every third day over 7 days, see Methods). The best induction of both CD4^+^ and CD8^+^ Foxp3^+^ Tregs was obtained with daily injections of 1 mg NPs, with a peak expansion of CD4^+^ Tregs peaking at day 7 and at day 14 for CD8^+^ Tregs (**Figure 1**).

**Figure 2:**
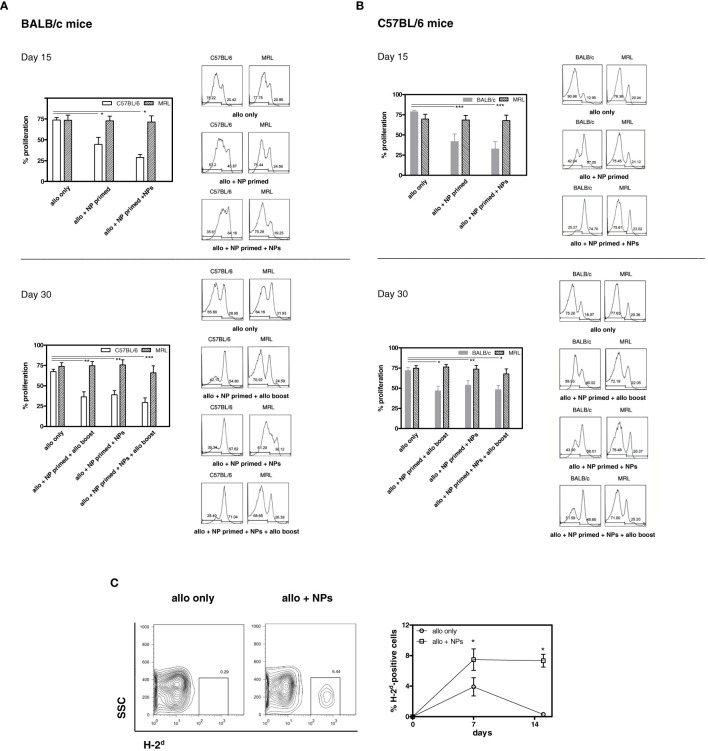
Tolerogenic NPs induce T cell allosuppression. **(A)** Allosuppression in BALB/c mice immunized with C57BL/6 splenocytes. *Top.* Cumulative and representative results of BALB/c T-cell proliferation to C57BL/6 and to MRL mouse splenocytes at day 15 day post-immunization (protocol in **Supplementary Table 1**). Measurement of CFSE staining by flow cytometry was assessed 72 h after coculture. *Bottom.* Cumulative and representative results (see top panel) at day 30 post-immunization show persistent allosuppression. Results are from three independent experiments with six mice/group; *P < 0.05, **<0.01, ***<0.005 by ANOVA vs. allotransplant only. **(B)** Allosuppression in C57BL/6 mice immunized with BALB/c splenocytes. *Top.* Cumulative and representative results of C57BL/6 T-cell proliferation to BALB/c and to MRL mouse splenocytes at day 15 day post-immunization (protocol in **Supplementary Table 1**). Measurement of CFSE staining by flow cytometry was assessed 72 h after coculture. *Bottom.* Cumulative and representative results (see top panel) at day 30 post-immunization show persistent allosuppression. Results are from three independent experiments with six mice/group; *P < 0.05, **<0.01, ***<0.001 by ANOVA vs. allotransplant only. **(C)** Allotransplanted cells persist in mice treated with tolerogenic NPs. PBMCs from C57BL/6 mice (H-2^b^) that received BALB/c (H-2^d^) splenocytes alone or together with tolerogenic NPs as per protocol (group 3 of **Supplementary Table 1**) were evaluated by flow cytometry for the expression of circulating H-2^d^-positive cells. Representative data at day 15 post-transfer of splenocytes (plots on the left) and cumulative data (right graph) in three mice/group in two independent experiments; *P < 0.005.

At these time points, the frequency of CD4^+^ Tregs doubled from baseline frequency, whereas CD8^+^ Tregs had a fourfold increase. The expansion was specific for Tregs since treatment with tolerogenic NPs did not affect the overall frequencies of T cells (**Supplementary Figure 2**). The frequencies of both CD4^+^ and CD8^+^ Tregs returned to baseline levels by day 21 (**Figure 1**). While follow-up studies will need to clarify the reason(s) for the sequential peak expansion of CD4^+^ vs. CD8^+^ Tregs after treatment, the results indicated that daily injections of NPs were most effective in expanding Tregs. With this information, we set up a protocol of NP delivery (**Supplementary Table 1** **).** **Figure 2** shows one of three representative experiments (eight mice/group each) that indicated that treatment with tolerogenic NPs inhibited immunization to alloantigen while maintaining a normal immunogenic response to third-party alloantigens (MRL mice). Specifically, **Figure 2A** shows the effects of NPs in allotransplantation of C57BL/6 splenocytes into BALB/c mice. At day 15, there was a strong allogeneic MLR of BALB/c mice (H2^d^) to C57BL/6 (H2^b^) and to third-party control MRL (H2^k^) mice. However, in BALB/c mice primed with tolerogenic NPs, MLR alloreactivity to C57BL/6 was significantly lower and was further reduced in NP-primed mice that had received additional doses of tolerogenic NPs. By contrast, alloreactivity to third-party controls remained strong. At day 30, the baseline MLR responses persisted to both C57BL/6 and MRL (third-party control mice) stimulator cells. However, NP-primed BALB/c mice—including those that had received additional NPs or allosplenocyte boost on day 24—had significantly lower MLR to C57BL/6 stimulator cells. The finding of a higher decrease in BALB/c mice that had received both NPs and a C57BL/6 splenocyte boost suggests a possible favorable role of alloantigen stimulation on MLR suppression. In any case, specificity was confirmed by the finding of MLR responses to the third-party MRL control mice remained strong during the entire course of the experiments despite a decreased responsiveness to C57BL/6 stimulator cells (**Figure 2A** **).** Similar inhibitory effects of the tolerogenic NPs on alloreactivity were observed when C57BL/6 mice were immunized with BALB/c splenocytes (**Figure 2B**). On day 15, there was a marked inhibition of MLR in C57BL/6 mice that had been NP-primed, and even more prominently in the mice receiving repeated administration of NPs. As with the BALB/c mice, the strength of the inhibition of the alloreactive response by the NPs was indicated by no further decrease of MLR responses following a boost with allogeneic splenocytes (**Figure 2B** **).** Although at day 30 the administration of NPs did not result in inhibition of alloreactivity as strong as at day 15, it remained nonetheless lower. Of note, the allotransplanted cells remained in the peripheral blood of NP-treated mice but not in controls (**Figure 2C**), validating the decreased MLR reactivity and suggesting achievement of a tolerogenic response.

### Effects of tolerogenic NPs on Tregs in alloimmunization

**Figure 3:**
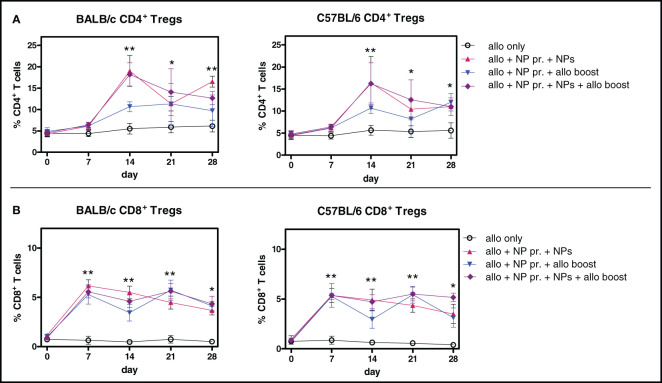
Alloimmunization together with treatment with tolerogenic NPs induces CD4^+^ and CD8^+^ Tregs. Percentages of circulating Tregs in mice during the experimental period. The legends on the right indicate the experimental conditions of the groups. Results are from three independent experiments with 6 mice/group. **(A)** CD4^+^ Tregs **(B)** CD8^+^ Tregs. *P < 0.05; **<0.01 vs. allotransplant only.

The inhibition of the alloreactive responses induced by tolerogenic NPs (**Figures 2A, B**) associated with the expansion of both CD4^+^ and CD8^+^ Foxp3^+^ Tregs (**Figure 3**) but not of CD4^+^ and CD8^+^ T cells *in toto* at any time point (data not shown). Mice receiving allogenic splenocytes (**Supplementary Figure 3**), when treated with tolerogenic NPs, had the frequency of CD4^+^ Tregs increased by fourfold (BALB/c mice) or threefold (C57BL/6 mice) by day 14, including when continuously receiving NPs (**Figure 3A**). For the CD8^+^ Tregs—although fewer than the CD4^+^ Tregs—a fivefold increase was observed by day 7 after NP administration, persisting throughout a 28-day follow-up (**Figure 3B** **).**

### Effects of tolerogenic NPs on dendritic cells in alloimmunization

It is known that CD4^+^ Foxp3^+^ Tregs not only suppress the activity of effector T and B cells but also inhibit immature DCs from becoming immunogenic DCs (18). Tregs can also induce DCs to become tolerogenic and further expands Tregs (20, 21).

**Figure 4:**
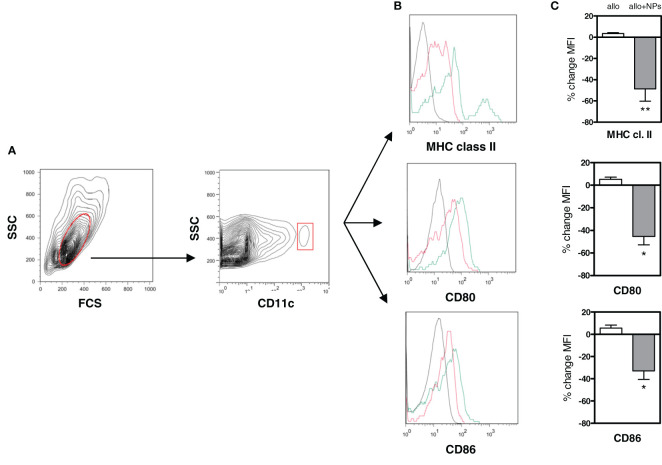
Tolerogenic NPs in allotransplantation promote a tolerogenic phenotype in dendritic cells. DCs from C57BL/6 mice allotransplanted with BALB/c splenocytes alone or together with NP treatment (group 3 of **Supplementary Table 1**) were analyzed *ex vivo* by flow cytometry at day 15 after start of treatment. **(A)** Gating of DCs (in red) from PBMCs as CD11c^+^ cells. **(B)** Representative histograms of cell surface staining for MHC class II, CD80, and CD86 on the gated DCs. Red lines indicate DCs from alloimmunized mice receiving NPs; green lines from mice alloimmunized only (no NPs). Black lines are isotype controls. **(C)** Cumulative percentage changes in mean fluorescence intensity (MFI) of the DC markers assessed in panel **(B)** Results are from two independent experiments with 6 mice/group; *P < 0.01; **<0.05.

Importantly, the DCs from NP-treated mice did induce Tregs directly, since coincubation of the NP-treated mice DCs with only T cells induced CD4^+^ and CD8^+^ Tregs (**Supplementary Figure 4**). To evaluate the effects of the NPs on DCs, the cell surface expression of molecules associated with a tolerogenic DC phenotype (21, 22) was assessed by flow cytometry on recipient mouse peripheral DCs. A marked decrease in the expression of MHC class II, CD80, and CD86 on the DCs from NP-treated mice (**Figure 4**) suggested the induction of a tolerogenic phenotype in DCs.

## Discussion

We have previously reported the development of a nanoproduct that protected mice from lupus-like disease and GvHD by inducing multiple tolerogenic immune cell populations *in vivo (* 14, 16, 17) and that inhibited T follicular helper (Tfh) cells (23). Here, we evaluated the effects of this nanoproduct in allotransplantation.

It has been reported that allospecific Tregs generated *ex vivo* can extend the survival of MHC-mismatched heart transplants in mice (24). Confirming past observations (25), those studies also showed that continuous allogeneic stimulation promoted the induction of CD4^+^ Tregs that maintained transplantation tolerance. Here, we show that it is possible to induce and maintain tolerogenic responses to alloantigen in two MHC-mismatched mouse strains via treatment with tolerogenic NPs. Specifically, we found 1) a marked decrease in T-cell proliferation to mismatched alloantigen; 2) the expansion of both CD4^+^ and CD8^+^ Tregs, 3) an induction of tolerogenic DCs, and 4) the presence of non-rejected allogeneic cells in the peripheral blood of NP-treated mice. Importantly, there was alloantigen specificity because the MLR to third-party alloantigen was maintained.

It has to be noted that although the NP-induced tolerogenic effects were observed in both BALB/c and C57/BL6 mice, the finding of a stronger protection in BALB/c mice suggests the possibility that the allelic makeup of the host might be critical (as seen in kidney and corneal graft allotransplants (26)) for a proper dosage of the NPs to induce transplant tolerance. Another consideration is that the NP-induced CD8^+^ Tregs and CD8^+^ Tregs are important contributors to the maintenance of transplant tolerance (27, 28). Although the frequency of the CD4^+^ Tregs was significantly higher, the concomitant expansion of the CD8^+^ Tregs might confer an advantage by virtue of recognizing MHC class I-restricted antigens— which are present on all cell types rather than on only MHC class II-expressing cells (29). Other possible benefits deriving from the expansion of the CD8^+^ Tregs could be the targeting of not only donor graft cells but also DCs (30), and the synergistic yet complementary activity with the CD4^+^ Tregs (31–33). Although these possibilities should be investigated directly, the current results on Tregs and DCs suggest both direct and indirect effects of the NPs on the generation of alloantigen specific Tregs. PLGA NPs loaded with peptides have been reported to migrate to tolerogenic organ(s) such as the liver, where they can promote generation of Tregs (34) which not only are immunosuppressive but also interact with DCs (18, 21), promoting tolerogenicity (20).

In sum, the herein reported use of NPs may represent a new modality in transplantation tolerance to switch immunogenic responses to alloantigen to tolerogenic responses. Compared with traditional therapies, NPs have the advantage of reducing undesired side effects and increase safety because of the delivery of two–three log lesser amounts of therapeutic agents used under conventional systemic administration (15).

Further studies will need to define the individual effects on allograft survival of the NP-induced Tregs and DCs and whether immune suppression will or will not be required to sustain the maintenance of the graft without persistent alloantigen stimulation.

## Data availability statement

The original contributions presented in the study are included in the article/**Supplementary material**. Further inquiries can be directed to the corresponding authors.

## Ethics statement

The animal study was approved by Animal Research Committee of the University of California Los Angeles. The study was conducted in accordance with the local legislation and institutional requirements.

## Author contributions

DH: Conceptualization, Data curation, Formal analysis, Methodology, Project administration, Supervision, Validation, Visualization, Writing – original draft, Writing – review & editing, Funding acquisition. JW: Writing – review & editing, Data curation, Formal analysis, Investigation, Software, Validation. DK: Writing – review & editing, Resources. CK: Resources, Writing – review & editing. KB: Writing – review & editing, Formal analysis, Investigation, Software, Validation. SB: Writing – review & editing, Methodology, Visualization. AC: Methodology, Visualization, Writing – review & editing, Conceptualization, Data curation, Formal analysis, Investigation, Project administration, Resources, Supervision, Validation, Writing – original draft.

## Supplementary material

Supplementary Table 1 Experimental protocol. C57BL6 or BALB/c mice served as either donors or recipients of the other strain’s splenocytes. All mice were injected with allogeneic splenocytes on day 3. Five groups were studied. Group 1 (allo only) also received a boost of allogeneic cells on day 24. Group 2 (NP-primed) also received NPs daily from day 0 to day 6. Group 3 (NP-primed + NPs) received NPs daily from day 0 to day 6 and then every three days thereafter until day 27. Group 4 (NP-primed + allo boost) received NPs daily from day 0 to day 6 and a boost with allogeneic cells on day 24. Group 5 (NP-primed + NPs + allo boost) received NPs daily from day 0 to day 6 and then every 3 days thereafter until day 27 plus a boost with allogeneic cells on day 24. MLRs with third party control MRL mouse splenocytes were performed on days 15 and 30. Tregs were identified by flow cytometry on days 7, 14, 21, and 28 by flow cytometry. The phenotype of dendritic cells was determined by flow cytometry on day 14.

Supplementary Figure 1 Schematic diagram of the procedure of assembly of the tolerogenic NPs used in the study.

Supplementary Figure 2 Daily i.p. injections with 1 mg anti-CD2 Ab-coated NPs containing IL-2 and TGF-β NPs do not alter the frequency of peripheral CD4^+^ and CD8^+^ T cells. Representative flow cytometry data on PBMCs on day 10.5 from the start of treatment from two independent experiments with 6 mice/group, *P* ns. Left panels: gating for lymphocytes; right panels: staining for CD4 and CD8. A PBMCs after treatment with PBS. B. PBMCs after treatment with NPs.

Supplementary Figure 3 Expansion of allo-Tregs after transplant. Representative flow cytometry staining for CD4^+^ Tregs in naïve BALB/c mice (n = 3) receiving autologous splenocytes (left) vs. allogeneic C57BL/6 splenocytes (right).

Supplementary Figure 4 Dendritic cells (DCs) from NP-treated mice directly induce CD4^+^ and CD8^+^ Tregs. Mice (n = 3) that had been treated with NPs (group 3 of **Supplementary Table 1**) or PBS were used as donors of DCs on day 15 after treatment. Untouched DCs purified from spleens using the Miltenyi Biotec Pan Dendritic Cell Isolation kit were cocultured with magnetic bead-sorted syngeneic CD25-depleted CD3^+^ T cells at a 1:4 ratio at 37°C/5% CO_2_. The figure shows representative flow cytometry plots of two independent experiments at day 3 after culture that indicate an expansion of CD4^+^ Tregs (top) and CD8^+^ Tregs (bottom) when DCs were from NP-treated animals.
