# Supplementary material for: Nanoparticles loaded with IL-2 and TGF-β promote transplantation tolerance to alloantigen
Source: Front Immunol. 2024 Jul 26;15:1429335. doi: 10.3389/fimmu.2024.1429335 (PMC11310063; doi:10.3389/fimmu.2024.1429335)
Supplement: Supplementary Table 1 — Experimental protocol. C57BL6 or BALB/c mice served as either donors or recipients of the other strain’s splenocytes. All mice were injected with allogeneic splenocytes on day 3. Five groups were studied. Group 1 (allo only) also received a boost of allogeneic cells on day 24. Group 2 (NP-primed) also received NPs daily from day 0 to day 6. Group 3 (NP-primed + NPs) received NPs daily from day 0 to day 6 and then every three days thereafter until day 27. Group 4 (NP-primed + allo boost) received NPs daily from day 0 to day 6 and a boost with allogeneic cells on day 24. Group 5 (NP-primed + NPs + allo boost) received NPs daily from day 0 to day 6 and then every 3 days thereafter until day 27 plus a boost with allogeneic cells on day 24. MLRs with third party control MRL mouse splenocytes were performed on days 15 and 30. Tregs were identified by flow cytometry on days 7, 14, 21, and 28 by flow cytometry. The phenotype of dendritic cells was determined by flow cytometry on day 14. [file DataSheet_1.pdf]

Nanoparticles loaded with IL-2 and TGF-β promote transplantation tolerance to alloantigen

David A. Horwitz, Ju Hua Wang, Dongin Kim, Chang Kang, Katja Brion, Sean Bickerton, Antonio La Cava

Supplementary Data

Day of treatment	0	1	2	3	4	5	6	9	12	15	18	21	24	27	30
Group 1 (mock)				allo									allo		
Group 2	NP	NP	NP	allo+NP	NP	NP	NP								
Group 3	NP	NP	NP	allo+NP	NP	NP	NP	NP	NP	NP	NP	NP	NP	NP	
Group 4	NP	NP	NP	allo+NP	NP	NP	NP						allo		
Group 5	NP	NP	NP	allo+NP	NP	NP	NP	NP	NP	NP	NP	NP	allo+NP	NP	
MLR										x					x
Flow cytometry							x			x		x		x	

Supplementary Table 1. Experimental protocol. C57BL6 or BALB/c mice served as either donors or recipients of the other strain's splenocytes. All mice were injected with allogeneic splenocytes on day 3. Five groups were studied. Group 1 (allo only) also received a boost of allogeneic cells on day 24. Group 2 (NP-primed) also received NPs daily from day 0 to day 6. Group 3 (NP-primed + NPs) received NPs daily from day 0 to day 6 and then every three days thereafter until day 27. Group 4 (NP-primed + allo boost) received NPs daily from day 0 to day 6 and a boost with allogeneic cells on day 24. Group 5 (NP-primed + NPs + allo boost) received NPs daily from day 0 to day 6 and then every 3 days thereafter until day 27 plus a boost with allogeneic cells on day 24. MLRs with third party control MRL mouse splenocytes were performed on days 15 and 30. Tregs were identified by flow cytometry on days 7, 14, 21, and 28 by flow cytometry. The phenotype of dendritic cells was determined by flow cytometry on day 14.

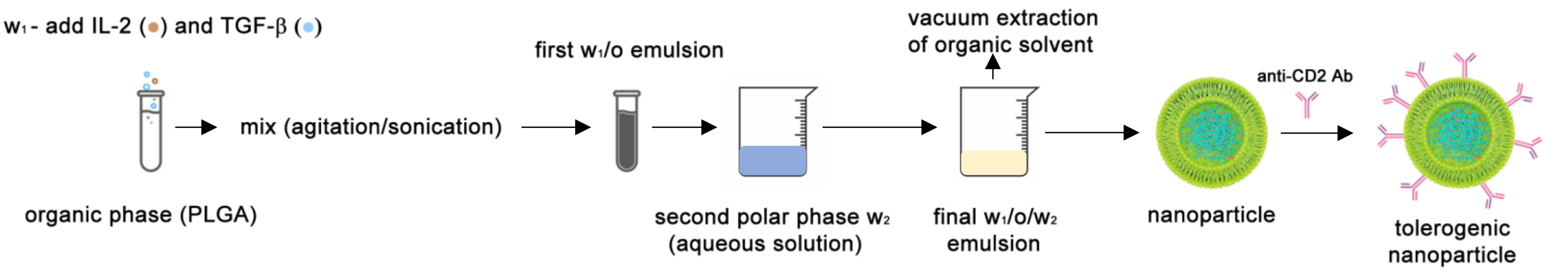

Supplementary Figure 1. Schematic diagram of the procedure of assembly of the tolerogenic NPs used in the study.

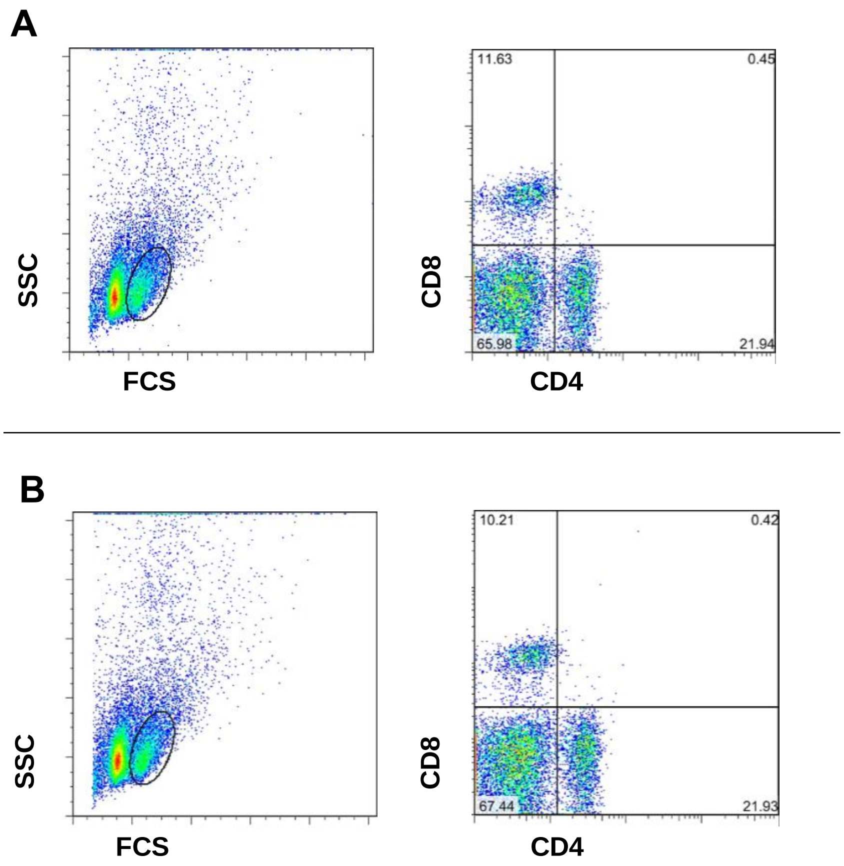

Supplementary Figure 2. Daily i.p. injections with 1 mg anti-CD2 Ab-coated NPs containing IL-2 and TGF-β NPs do not alter the frequency of peripheral CD4$^+$ and CD8$^+$ T cells. Representative flow cytometry data on PBMCs on day 10.5 from the start of treatment from two independent experiments with 6 mice/group, *P* ns. Left panels: gating for lymphocytes; right panels: staining for CD4 and CD8. A PBMCs after treatment with PBS. B. PBMCs after treatment with NPs.

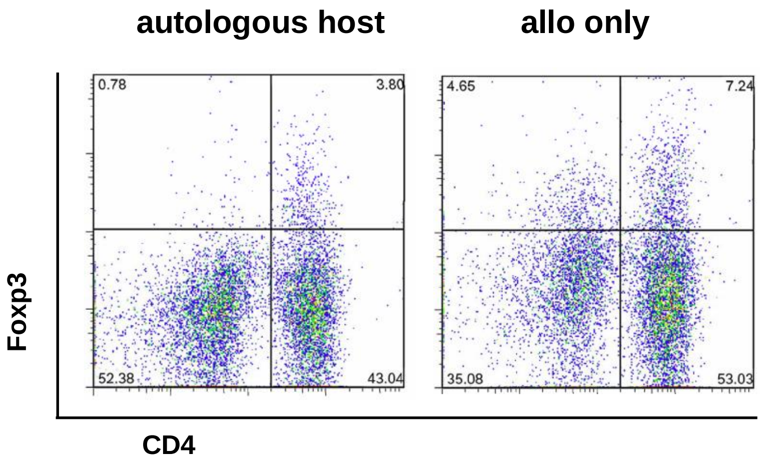

Supplementary Figure 3. Expansion of allo-Tregs after transplant. Representative flow cytometry staining for $CD4^+$ Tregs in naïve BALB/c mice (n = 3) receiving autologous splenocytes (left) vs. allogeneic C57BL/6 splenocytes (right).

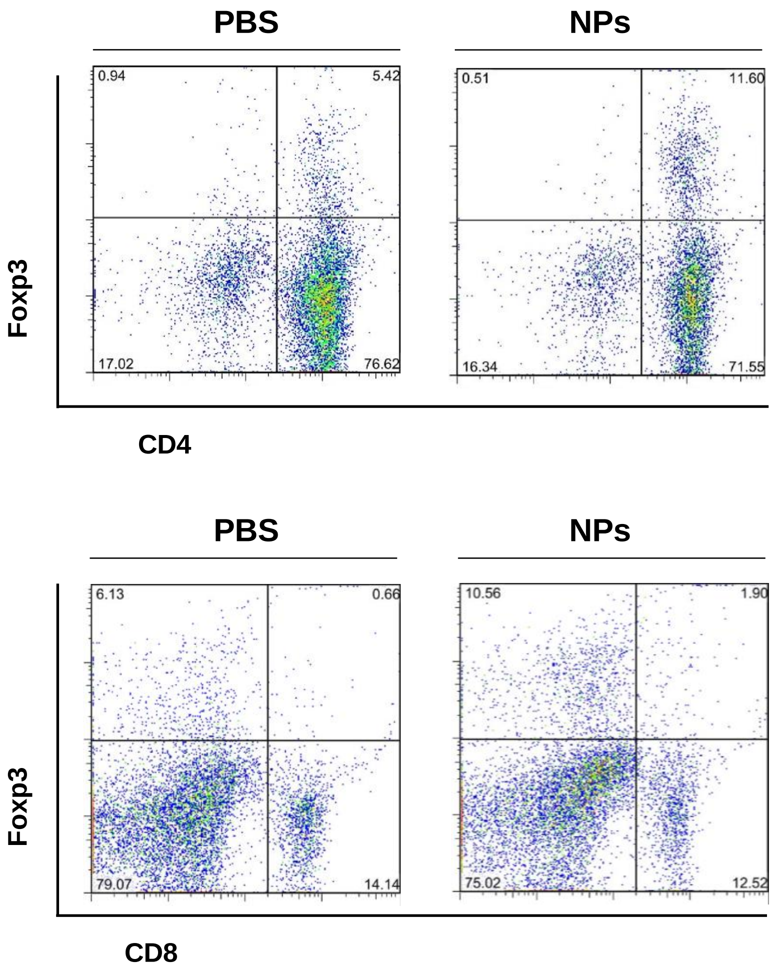

Supplementary Figure 4. Dendritic cells (DCs) from NP-treated mice directly induce CD4⁺ and CD8⁺ Tregs. Mice (n = 3) that had been treated with NPs (group 3 of Supplementary Table 1) or PBS were used as donors of DCs on day 15 after treatment. Untouched DCs purified from spleens using the Miltenyi Biotec Pan Dendritic Cell Isolation kit were cocultured with magnetic bead-sorted syngeneic CD25-depleted $CD3^+$ T cells at a 1:4 ratio at 37°C/5% CO_2. The figure shows representative flow cytometry plots of two independent experiments at day 3 after culture that indicate an expansion of $CD4^+$ Tregs (top) and $CD8^+$ Tregs (bottom) when DCs were from NP-treated animals.
